# Weight Change and Accumulation of Chronic Conditions in Women During Reproductive Years

**DOI:** 10.1002/oby.70023

**Published:** 2025-09-02

**Authors:** Mohammad R. Baneshi, Annette Dobson, Gita D. Mishra

**Affiliations:** ^1^ Australian Women and Girls' Health Research Centre, School of Public Health The University of Queensland Herston Queensland Australia

**Keywords:** BMI, multimorbidity, reproductive age, weight change, weight control

## Abstract

**Objective:**

We examined the association between BMI change and risk of multimorbidity among women of reproductive age and estimated annual weight gain before and after diagnosis of chronic conditions.

**Methods:**

Data were from 8895 women in the Australian Longitudinal Study on Women's Health who had no chronic conditions at baseline. BMI from Survey 1 (ages 18–23) and Survey 3 (ages 25–30) defined BMI change categories. Linear mixed models estimated annual weight gain from Survey 1 to Survey 9 (ages 43–49).

**Results:**

Compared to stable normal BMI, stable obesity and increasing BMI were associated with a higher risk of incident multimorbidity (OR = 1.79 [95% CI: 1.11, 2.90] and 1.34 [1.10, 1.63]). Women who remained free of chronic conditions gained 0.50 kg (0.48, 0.52) annually. Women with one condition gained 0.54 kg (0.52, 0.56): 0.55 (0.53, 0.57) before and 0.54 (0.51, 0.57) after diagnosis. Those with multimorbidity gained 0.65 kg (0.63, 0.67): 0.75 kg (0.70, 0.80) before the first, 0.60 kg (0.56, 0.64) between the first and second, and 0.57 kg (0.53, 0.61) after the second condition.

**Conclusions:**

Although weight gain declined after diagnosis, it remained higher than among women without conditions, underscoring the need for improved post‐diagnosis weight management.

**Study Importance**

- What is already known?
  ○ Longitudinal studies have established an association between change in BMI category and the subsequent risk of chronic diseases or multimorbidity.
  ○ No study has investigated how weight gain varies during different phases of disease accumulation.
- What does this study add?
  ○ This study focused on 8895 women from the Australian Longitudinal Study on Women's Health who were free of chronic conditions at the baseline survey. This research estimated annual weight gain overall and before and after the diagnosis of chronic conditions.
  ○ Over 25 years, women who remained free of chronic conditions gained an average of 0.50 kg per year (95% CI: 0.48, 0.52). Women who developed a single condition gained 0.54 kg (0.52, 0.56) annually: 0.55 (0.53, 0.57) before and 0.54 (0.51, 0.57) after diagnosis. Those with multimorbidity experience the highest weight gain of 0.65 kg (0.63, 0.67) annually: 0.75 kg (0.70, 0.80) before the first condition, 0.60 kg (0.56, 0.64) between the first and second, and 0.57 kg (0.53, 0.61) following the second condition.
- How might these results change the direction of research or the focus of clinical practice?
  ○ Women diagnosed with chronic conditions experienced a slowdown in annual weight gain; however, their weight gain still exceeded that of women without any chronic condition. This highlights the need for effective preventive strategies and improved weight management following the diagnosis of chronic conditions.

## 1 Introduction

High body mass index (BMI) (i.e., having overweight or obesity) has become a major epidemic in the 21st century [1]. According to the World Health Organization (WHO), in 2022, 59% of adults were classified as having overweight or living with obesity [2]. At the same time, multimorbidity—defined as having two or more chronic conditions in the same individual—has emerged as another pressing health challenge. The consequences of multimorbidity include poorer health outcomes [3], higher use of health services [4], and more frequent hospitalization [5].

High BMI is a well‐documented risk factor for numerous chronic conditions including stroke [6], coronary heart disease [7], and diabetes [8]. Furthermore, high BMI is a key contributor to multimorbidity [9, 10, 11, 12], with a recent systematic review indicating a dose–response association, showing that each unit increase in BMI is associated with a 6.0% increase in the risk of multimorbidity [12]. Longitudinal studies have further established an association between weight change (or change in BMI category) and the subsequent risk of chronic diseases or multimorbidity [13, 14, 15, 16, 17].

Women of reproductive age are particularly impacted by both high BMI and multimorbidity. Maternal obesity is increasingly prevalent, affecting 16.3% of pregnant women globally, with a combined prevalence of overweight and obesity at 43.8% [18]. Obesity in women is associated with adverse reproductive health outcomes including menstrual irregularity and reduced fertility [19], as well as increased risks of gynecological chronic conditions like endometriosis and uterine fibroids [20, 21]. Moreover, women are at higher risk of multimorbidity compared to men, often with earlier onset [22].

While the association between weight change and multimorbidity is well established, most existing studies have focused on elderly populations of both men and women [14, 17]. Women of reproductive age represent a crucial yet understudied population, despite the impact this life stage has on their immediate and long‐term health. Additionally, to our knowledge, no study has investigated how weight gain varies during different phases of disease accumulation. The Australian Longitudinal Study on Women's Health (ALSWH) provides a unique opportunity to investigate patterns of weight change among women of reproductive age. Specifically, this study aims to (a) compare the average weight before and after the diagnosis of individual chronic conditions, (b) estimate the average annual weight gain during different phases of the accumulation of multiple chronic conditions, before and following their onset, and (c) investigate the association between weight change and the subsequent incidence of multimorbidity.

## 2 Methods

### 2.1 Study Population

The ALSWH is a prospective, nationwide, population‐based study of four cohorts born in 1989–95, 1973–78, 1946–51, and 1921–26 [23]. The three oldest cohorts were randomly selected from all women in the Medicare database, which is the database of the national universal health insurance scheme that covers all citizens and permanent residents in Australia.

The current analyses are related to women born in 1973–78 who consented to data linkage [24]. The first survey was conducted in 1996 (ages 18–23 years); followed by the second in 2000. Subsequent surveys were conducted every 3 years, with the latest survey (i.e., Survey 9) completed in 2021 (ages 43–49).

### 2.2 Ethical Approval

The ALSWH has been granted ethics clearance by the Human Research Ethics Committees at the University of Newcastle (ref no. H‐0760‐0795) and the University of Queensland (ref no. 2004000224). Details of ALSWH ethical approvals for linked administrative datasets are provided here: https://alswh.org.au/alswh‐hrec‐approvals/.

This study has been approved by the University of Queensland (Project ID: A1392). Informed consent was obtained from participants for each survey. All participants consented to data linkage. All methods were carried out in accordance with relevant guidelines and regulations. The authors declare that this work was in compliance with ethical standards.

### 2.3 List of Chronic Conditions

In ALSWH Surveys 1 and Survey 2, women were asked, “Have you ever been told by a doctor that you have …?” followed by a list of specific conditions. From Survey 3 onward, the question was changed to “In the past 3 years, have you been diagnosed or treated for …?” and was also accompanied by a list of conditions. These survey data were linked to administrative health records including hospital admissions; investigations, procedures, and medication prescriptions subsidized by the national health insurance scheme; assessments for aged care support; and medically certified causes of death. The sources of data used to identify women with each chronic condition and the coverage period of each data source are summarized in Table S1.

Our list of conditions included the following 12 chronic conditions: endometriosis; uterine fibroids; poor mental health (including only depression or anxiety); musculoskeletal disorders (including back pain, rheumatoid arthritis, osteoarthritis, ankylosing spondylitis, cervical disc displacement, sciatica, spinal deformities, and scoliosis); asthma; cancer (including malignant cancers but not skin cancer except for melanoma); diabetes (including type 1 and 2 diabetes mellitus and excluding gestational diabetes); heart disease (including heart surgery and interventions [heart bypass, angioplasty, and angiography] and acute coronary syndrome but not heart failure); stroke (including ischemic and hamorrhagic stroke); eating disorders (including anorexia and bulimia); chronic obstructive pulmonary disease (COPD); and dementia (included Alzheimer's dementia, vascular dementia, and unspecified dementia).

### 2.4 Study Sample

**FIGURE 1:**
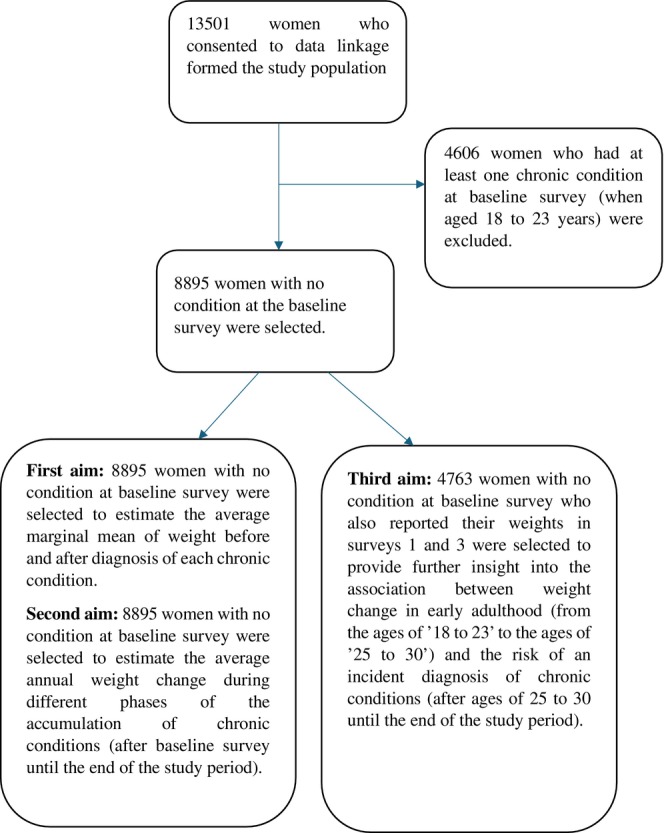
Flowchart of selection of participants for different analyses.

Of the 14,247 women who participated in the first ALSWH survey, 13,501 women consented to data linkage and were eligible for inclusion. Of these 13,501 women, 8895 had no chronic condition at the baseline survey and formed the study sample (Figure 1).

### 2.5 Statistical Analysis

For 13,501 women who consented to data linkage, the progression of each of the 12 chronic conditions at Survey 1 and by the end of the study period (i.e., Survey 9) was described. Moreover, for 8895 women with no chronic conditions at the baseline survey, the progression of conditions after Survey 1 and up to the end of the study period was described.

#### 2.5.1 First Aim: Comparing the Average Weight Before and After Diagnosis of Each Chronic Condition (*N* = 8895)

For the first and second aims, 8895 women with no chronic conditions at the baseline survey formed the sample (Figure 1). We examined the association between the time‐dependent effect of individual chronic conditions (i.e., exposure variables) and weight (i.e., outcome) using a linear mixed model. To reduce noise from rare conditions, only those with a prevalence above 2.0% were included. The analysis was adjusted for the time‐dependent effect of smoking status, the ability to manage on income, physical activity, number of live births, and menopause status. To account for regression to the mean, baseline BMI in continuous form was included in the model. For each condition, the estimated marginal mean of weight before and after diagnosis, along with the mean difference and corresponding 95% confidence interval (CI), was estimated.

#### 2.5.2 Second Aim: Estimating the Annual Weight Gain During Different Phases of Accumulation of Chronic Conditions (*N* = 8895)

A linear mixed regression model was used to model weight as a function of age. The coefficient of age was interpreted as the average annual weight gain. We created a variable that tracked the cumulative number of chronic conditions reported by each subject at each survey. An interaction term between age and the number of chronic conditions was included in the model to investigate whether average weight gain varies during different phases of the accumulation of chronic conditions.

To examine the correlation structure between repeated measurements of weight, the Pearson correlation was calculated for all pairs of weights across the nine surveys. Correlations were found to be higher for surveys conducted closer in time and diminished as the time gap between surveys increased. Therefore, to capture the pattern of declining correlation with increasing time between surveys, an autoregressive correlation structure was used to model the dependency of observations within each individual. The analyses were adjusted for the same set of covariates.

#### 2.5.3 Third Aim: Association Between Change in BMI Category From Survey 1 to Survey 3 and Incidence of Multimorbidity (*N* = 4763)

In each ALSWH survey, participants were asked to report their weight and height. BMI was calculated based on self‐reported height and weight in each of the nine ALSWH surveys. Applying cutoff values of 18.5, 24.9, and 29.9 kg/m^2^, women were classified in each survey into the categories of underweight, normal, overweight, or obesity.

Comparing BMI categories in Survey 1 (ages 18–23) and Survey 3 (ages 25–30), women were divided into five BMI change categories: women with a stable normal weight (including stable underweight, stable normal, or transition from underweight to normal); women with stable overweight; those with stable obesity; those with increasing BMI; and those with decreasing BMI. Therefore, for this analysis, out of 8895 women with no chronic conditions at the baseline survey, 4763 women who reported their weight and height in Survey 1 and Survey 3 were included (Figure 1).

The outcome was incident multimorbidity—defined as the reporting of two or more chronic conditions after Survey 3 (i.e., after ages 25–30) up to and including Survey 9 (ages 43–49). A logistic regression model was fitted to estimate the odds ratio (OR) of incident multimorbidity, with the women with a stable normal category serving as the reference category. The analyses were adjusted for the baseline effect of smoking status, the ability to manage on income, physical activity, number of live births, and BMI (in continuous form).

### 2.6 Software

Data were analyzed using R software with the following packages: “tidyverse” for data management, “ggplot2” for graphical visualizations, and “nlme” for mixed‐effects regression models.

## 3 Results

For women who consented to data linkage (*N* = 13,501), at the baseline survey in 1996 (when they were ages 18– 23 years), the most prevalent chronic conditions were poor mental health (21.7%), musculoskeletal disorders (10.1%), and asthma (7.9%) (Table 1). By the end of the study (ages 43–49), the most prevalent chronic conditions were poor mental health (51.3%), musculoskeletal disorders (42.6%), and endometriosis (13.7%). The proportion of women with no chronic condition decreased from 65.9% at baseline to 25.4% at the end of the study period (Table 1). Conversely, the proportion of women with multimorbidity increased from 6.5% at baseline to 41.8% at the end of the study.

**TABLE 1:** Progression of chronic conditions over time in a cohort of Australian women born between 1973 and 1978.

Subgroup	All women who consented to data linkage (*N* = 13,501)		Women with no chronic conditions at baseline survey (*N* = 8895)
Period over which proportions are reported	By Survey 1 (ages 18–23 years)	By the end of the study (ages 43–49 years)	After Survey 1 until the end of the study
Poor mental health	2934 (21.7%)	6924 (51.3%)	3146 (35.4%)
Musculoskeletal	1370 (10.1%)	5758 (42.6%)	2988 (33.6%)
Endometriosis	37 (0.3%)	1844 (13.7%)	1067 (12.0%)
Asthma	1071 (7.9%)	1552 (11.5%)	308 (3.5%)
Uterine fibroids	0 (0.0%)	751 (5.6%)	459 (5.2%)
Cancer	34 (0.3%)	647 (4.8%)	395 (4.4%)
Diabetes	71 (0.5%)	723 (5.4%)	360 (4.0%)
Heart disease	9 (0.1%)	223 (1.7%)	120 (1.3%)
Stroke	0 (0.0%)	115 (0.9%)	60 (0.7%)
Eating disorder	52 (0.4%)	108 (0.8%)	27 (0.3%)
COPD	0 (0.0%)	68 (0.5%)	20 (0.2%)
Dementia	0 (0.0%)	1 (0.0%)	1 (0.0%)
Number of conditions			
0	8895 (65.9%)	3427 (25.4%)	3427 (38.5%)
1	3724 (27.6%)	4429 (32.8%)	2985 (33.6%)
≥ 2	882 (6.5%)	5645 (41.8%)	2483 (27.9%)

Abbreviation: COPD: chronic obstructive pulmonary disease.

Among women who reported no condition at the baseline survey (*N* = 8895), the most prevalent conditions by the end of the survey were poor mental health (35.4%), musculoskeletal disorders (33.6%), and endometriosis (12.0%) (Table 1). The proportion of women in this group who developed multimorbidity after the baseline survey was 27.9%.

### 3.1 First Aim: Comparing the Average Weight Before and After Diagnosis of Each Chronic Condition (*N* = 8895)

The average weight increased following the diagnosis of poor mental health (1.0 kg; 95% CI: 0.8, 1.2), musculoskeletal disorders (0.6 kg; 95% CI: 0.4, 0.8), and asthma (1.4 kg; 95% CI: 0.7, 2.1) (Table 2). In contrast, weight decreased after a cancer diagnosis (−1.5 kg; 95% CI: −2.2, −0.8). For endometriosis, uterine fibroids, and diabetes, weight increases were small, and the CI for the differences included the null value of zero.

**TABLE 2:** Estimated average marginal mean weight (in kilograms) before and after diagnosis of each chronic condition.

Condition	Before diagnosis	After diagnosis	Difference (95% CI)
Poor mental health	69.0 (68.8, 69.2)	70.0 (69.8, 70.3)	1.0 (0.8, 1.2)
Musculoskeletal	69.2 (69.0, 69.4)	69.8 (69.4, 70.1)	0.6 (0.4, 0.8)
Endometriosis	69.3 (69.1, 69.5)	69.3 (68.8, 69.8)	0.0 (−0.3, 0.3)
Asthma	69.3 (69.1, 69.5)	70.7 (69.7, 71.6)	1.4 (0.7, 2.1)
Uterine fibroids	69.3 (69.1, 69.5)	69.5 (68.7, 70.2)	0.2 (−0.3, 0.5)
Cancer	69.3 (69.1, 69.5)	67.7 (66.9, 68.6)	−1.5 (−0.8, −2.2)
Diabetes	69.3 (69.1, 69.5)	69.7 (68.7, 70.6)	0.4 (−0.3, 1.1)

### 3.2 Second Aim: Estimating the Annual Weight Gain During Different Phases of Accumulation of Chronic Conditions (*N* = 8895)

**FIGURE 2:**
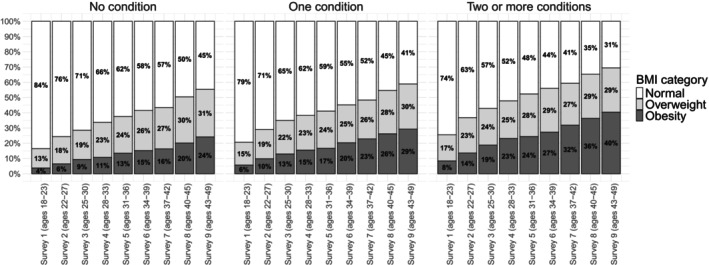
Distribution of BMI category across nine surveys for women with no chronic conditions at the baseline survey, stratified by the number of conditions at the end of the study. The normal weight category includes women whose BMI was in the normal or underweight category.

Figure 2 shows the distribution of the BMI category across nine surveys for women with no chronic conditions at the baseline survey, stratified by the number of chronic conditions developed by the end of the study. Compared to their counterparts who remained free of chronic conditions, women with multimorbidity exhibited a higher proportion in the overweight and obesity categories throughout all nine surveys. Furthermore, the disparity between women without chronic conditions and those with multimorbidity increased over time. For example, in Survey 1 (ages 18–23), the difference in obesity prevalence between these two groups was 4 percentage points (4% among women without chronic conditions vs. 8% among those with multimorbidity). By Survey 5 (ages 31–36) and Survey 9 (ages 43–48), this difference had expanded to 11 percentage points (13% vs. 24%) and 16 percentage points (24% vs. 40%) respectively.

**FIGURE 3:**
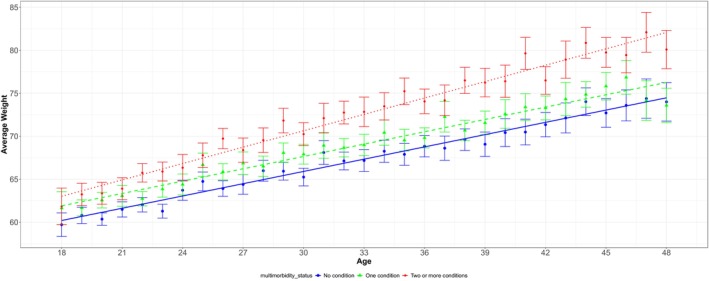
Observed average weight for women with no chronic conditions at the baseline survey, stratified by the number of chronic conditions at the end of the study. [Color figure can be viewed at wileyonlinelibrary.com]

Figure 3 shows the observed average weight with 95% CI across different ages, stratified by the number of chronic conditions at the end of the study period. Women reporting two or more chronic conditions had a higher average weight at the age of 18 compared to other women. The mean weight (SD) at age 18 was 59.7 kg (8.7) for women with no chronic conditions, 61.6 kg (11.8) for women with one, and 61.8 kg (12.0) for those who developed two or more chronic conditions throughout the study. At age 48, the corresponding figures were 74.0 kg (16.2), 74.0 kg (15.8), and 80.1 kg (18.9), respectively.

Women with no chronic conditions at the baseline survey who developed multimorbidity by the end of the study period experienced a greater average annual increase in weight compared to those with no chronic conditions or only one chronic condition at the baseline survey. The average annual weight gain from the baseline survey to the end of the study was 0.50 kg (95% CI: 0.48, 0.52) for women with no chronic conditions, 0.54 kg (0.52, 0.56) for those reporting one chronic condition, and 0.65 kg (0.63, 0.67) for women with multimorbidity (Table 3).

**TABLE 3:** Average annual weight gain for women with no chronic conditions at the baseline survey stratified by number of chronic conditions at the end of the study.

Number of chronic conditions at the end of the survey	Average annual weight gain in kilograms (95% CI)
0	0.50 (0.48, 0.52) from the baseline survey to the end of the study
1	0.54 (0.52, 0.56) from the baseline survey to the end of the study 0.55 (0.53, 0.57) from baseline to report of the first chronic condition 0.54 (0.51, 0.57) from the report of the first chronic condition to the end of the study
≥ 2	0.65 (0.63, 0.67) from the baseline survey to the end of the study 0.75 (0.70, 0.80) from baseline to the report of the first chronic condition 0.60 (0.56, 0.64) from the report of the first to the report of the second chronic condition 0.57 (0.53, 0.61) from the report of the second chronic condition to the end of the study

Among women who reported only one chronic condition, the average annual weight gain slightly declined from 0.55 kg (0.53, 0.57) (in the period from baseline to the report of a first chronic condition) to 0.54 kg (0.51, 0.57) (from the report of a first chronic condition to the end of the study) (Table 3).

For women with multimorbidity, the average annual gain was 0.75 kg (0.70, 0.80) from baseline to the report of the first chronic condition; 0.60 kg (0.56, 0.64) between the first and second chronic conditions; and 0.57 kg (0.53, 0.61) from the second chronic condition to the end of the study (Table 3).

### 3.3 Third Aim: Association Between Change in BMI Category and Incidence of Multimorbidity (*N* = 4763)

Transitions between BMI categories from Survey 1 to Survey 3 are summarized in Table S2. Of the 483 women classified as underweight in Survey 1, 59.6% had a normal BMI by Survey 3. Of the 3282 women with a normal BMI in Survey 1, 76.7% remained in the same category, 19.6% transitioned to the overweight category, and 3.7% moved to the obesity category. Among those classified as overweight in Survey 1, 38% (326 out of 720) transitioned to obesity, whereas 16.6% moved to the normal category. Additionally, 83.5% of women with obesity in Survey 1 remained in the obesity category in Survey 3 (232 out of 278).

The distribution of the number of chronic conditions (after Survey 3 until the end of the study) differed among women with different categories of BMI change. For example, 18.5% of women with stable normal BMI reported two or more chronic conditions, compared to 35.3% of those with stable obesity (Table 4). When compared to women with stable normal BMI, the OR for multimorbidity was 1.60 (95% CI: 1.05, 1.65) for women with stable obesity; 1.31 (95% CI: 1.06, 1.62) for women with increasing BMI, and 1.16 (95% CI: 0.80, 1.69) for women with decreasing BMI (Table 4).

**TABLE 4:** Association between change in BMI category and the subsequent risk of multimorbidity (*N* = 4763).

Change in BMI category	No condition	1 condition	≥ 2 conditions	OR of incident multimorbidity (95% CI)
Stable normal (*N* = 2916)	1379 (47.3%)	998 (34.2%)	539 (18.5%)	Reference group
Stable overweight (*N* = 326)	137 (42.0%)	121 (37.1%)	68 (20.9%)	0.93 (0.64, 1.33)
Stable obesity (*N* = 232)	69 (29.7%)	81 (34.9%)	82 (35.3%)	1.60 (1.05, 1.65)
Increasing BMI (*N* = 1049)	391 (37.3%)	398 (37.9%)	260 (24.8%)	1.31 (1.06, 1.62)
Decreasing BMI (*N* = 240)	100 (41.7%)	83 (34.6%)	57 (23.7%)	1.16 (0.80, 1.69)

*Note*: The weight change was determined by comparing BMI categories in Survey 1 and Survey 3. Women with no chronic conditions at the baseline survey, who reported their weight and height in Survey 1 and Survey 3 were included in this analysis.

Abbreviation: OR: odds ratio.

## 4 Discussion

While it is well established that weight gain (or change in BMI category) is associated with increased risk of multimorbidity, our study provides new insights by focusing specifically on women of reproductive age. Using nearly two decades of longitudinal data from a large population‐based sample of Australian women, we not only examined the association between change in BMI category and subsequent risk of multimorbidity but also estimated the average annual weight gain during different phases of disease accumulation.

First, we examined the effect of individual conditions on weight and found that cancer was associated with weight loss, whereas poor mental health, musculoskeletal disorders, and asthma were associated with weight gain. These results align with Jackson et al., who reported similar findings for cancer in two cohorts [25]. Other studies have also reported weight gain in relation to anxiety and depressive disorders [26], severe mental illness [27], and asthma [28].

By examining weight gain trajectories before and after the onset of multiple chronic conditions, our study provided a life‐course perspective that has been largely overlooked in the previous research. We found that women with multimorbidity experienced an average annual weight gain of 0.75 kg prior to the report of their first chronic condition. Notably, even after the report of a second chronic condition, their annual weight remained marginally higher than that of women without any chronic condition (0.57 vs. 0.50 kg).

Previous research reinforced the importance of the cumulative impact of small or modest weight gain. For example, 1 kg weight gain over 10 years (corresponding to 0.1 kg per year) was associated with a 7.3% increase in the risk of type‐2 diabetes [29]. Another study reported that weight gain of 2.5 to 10 kg over 18 years (corresponding to 0.1 to 1.8 kg annually) increased the risk of several chronic conditions, including type‐2 diabetes, cardiovascular disease, and obesity‐related cancer [17]. Among women in our cohort without chronic conditions, the average annual weight gain of 0.5 kg translated to a cumulative 12.5‐kg increase over 25 years. For those with multimorbidity, the gain was 0.65 kg annually, totaling about 16.5 kg over the same period. The difference of 4 kg between the two groups, equivalent to 1.5 BMI units for an average‐height woman of 1.65 m, could shift women into higher BMI categories and increase their risk for chronic disease.

Our study also found that women with stable obesity or increasing BMI were at a higher risk of being diagnosed with multimorbidity in later reproductive years. Conversely, the risk for women with a decreasing BMI did not differ from that of women with a stable normal BMI. These findings emphasize that reproductive years represent a critical window for women's overall health and future well‐being [30]. During this time, women are particularly vulnerable to weight‐related health issues that can impact both their reproductive function and general health [31]. By focusing on this life stage, our study builds on previous studies that have largely focused on older or mixed‐age populations, underscoring early adulthood as a pivotal period for preventing the progression of multimorbidity. The observed associations underscore the necessity for a life‐course approach to women's health, emphasizing the importance of both preventive strategies in early adulthood and targeted interventions following the onset of chronic conditions.

Several studies have investigated the association between weight change across a broad age range and the risk of developing new chronic conditions or multimorbidity [13, 14, 15, 16, 17]. These studies varied in the time frame during which weight change was assessed and the criteria used to classify the participants into different weight change categories. Nonetheless, all studies consistently found that people with an increasing weight pattern were at higher risk of new chronic conditions or multimorbidity. Zheng et al. conducted a cohort analysis involving 92,837 female registered nurses to investigate the association between weight change from ages 18 to 55 and the risk of developing a composite outcome measure of major chronic diseases (including type 2 diabetes, cardiovascular disease, cancer, and nontraumatic death after age 55) [17]. The analysis found a monotonically increasing association between weight gain exceeding 5 kg and increased risk of the composite outcome [17].

A similar study of 48,377 women (mean [SD] age 47.8 [5.3]) and 35,989 men (mean [SD] age 49.6 [5.1]) found that in both genders, a weight gain of 5 kg from early to midadulthood (ages 20–40 for women and 20–59 for men) was associated with an increased risk of all‐cause and cardiovascular disease‐related mortality [15].

Gong et al. analyzed data from 15,520 adults (49.5% women; mean [SD] age 70.0 [0.10]) who participated in the National Health and Nutrition Examination Survey in the United States. Participants were asked to report their weight at age 25 and also 10 years before the survey. Moreover, BMI was measured at the baseline survey during physical examination [14]. The primary outcome of the study was obesity‐related complex multimorbidity in later life, defined as the presence of two or more of the following conditions: hypertension, cancer, COPD, cardiovascular disease, and diabetes. When comparing BMI from 10 years before baseline with those at baseline, individuals with stable obesity exhibited higher odds of multimorbidity compared to those with a stable normal BMI. A similar conclusion was reached when comparing BMI at age 25 with baseline measurements. It has been concluded that maintaining a stable healthy weight during early adulthood and midlife is important for a better quality of life throughout the aging process [14].

Data from the 7357 women without a history of chronic conditions were used to investigate the effect of short‐term weight change with the accumulation of multimorbidity [16]. Women were surveyed every 3 years from 1996 (ages 45–50) to 2016 (ages 65–70). The short‐term weight change over 3‐year periods (except for 2 years between the first and second surveys) was calculated as the difference in weight between two consecutive surveys divided by the weight reported in the earliest survey and the number of years between the surveys. The analysis revealed that the odds of multimorbidity for women who experienced weight loss were similar to those for women with stable weight. Conversely, women who experienced small, moderate, or high gain of weight were found to be at an increased risk of multimorbidity.

Agborsangaya et al. posited that weight loss in patients with severe obesity may facilitate the remission of chronic conditions [13]. They conducted an analysis of a dataset comprising 500 adults with severe obesity (88.2% women; mean [SD] age 46.1 [9.5]) with BMI ≥ 35 to investigate the association between short‐term weight loss over a 2‐year period and the odds of reducing multimorbidity [13]. The number of chronic conditions was determined by counting 20 self‐reported chronic conditions. Participants were grouped into two categories based on whether the number of chronic conditions decreased over the 2‐year period. Compared to participants with < 5.0% weight loss, those with > 5% weight loss were 70.0% more likely to experience a reduction in the number of chronic conditions (OR = 1.70 [95% CI: 1.10, 2.70]).

On the other hand, a study focusing on older adults concluded that weight loss in older adults may be an indication of accelerated health deterioration [32]. Calderon‐Larranaga et al. investigated the association between a 12‐year BMI trajectory and the speed of accumulation of 60 chronic conditions among adults (63.0% women; mean [SD] age 71.6 [9.6]) [32]. Their findings demonstrated that the speed of accumulation of chronic conditions was higher among those with a fast‐decline BMI trajectory compared to those with a stable trajectory [32]. These results suggest that the association between BMI trajectory and the accumulation of chronic conditions may differ across different stages of life.

Our study had some limitations. First, there were differences in the periods when data were available for various chronic conditions (Table S1). Second, the diagnosis of chronic conditions was based on self‐report (from ALSWH surveys) and administrative health records. However, the use of linked administrative datasets represents a major strength of this study. As summarized in Table S1, multiple sources of data were used to identify reports of chronic conditions. Therefore, even if women were no longer completing the ALSWH surveys, the incidence of chronic conditions could be identified using other sources. Nevertheless, some cases may not have been reported. Moreover, it should be acknowledged that the ALSWH survey experienced moderate nonresponse rates during each follow‐up.

Previous studies supported the use of linked data for conditions such as cancer [33], diabetes, and stroke [34]. These findings highlight the limitations of relying solely on self‐report and support the inclusion of multiple data sources.

## 5 Conclusion

Our findings indicate that women who maintained obesity or experienced an increase in BMI category were at a higher risk of multimorbidity in later reproductive years. Moreover, while a diagnosis of chronic conditions slowed annual weight gain, it remains high compared to women without any chronic condition. Therefore, there is a need for more effective strategies and policies aimed at facilitating weight reduction following the diagnosis of chronic conditions.

## Author Contributions

All authors contributed to the design of the study. M.R.B. undertook the statistical analyses, and all authors interpreted the results. All authors read and revised the manuscript, accepted the final version of the manuscript, and were accountable for all aspects of the work.

## Conflicts of Interest

The authors declare no conflicts of interest.

## Supporting information


**Data S1:** Supporting Information.

## Data Availability

The data that support the findings of this study are available from Australian Commonwealth and State/Territory data custodians. Restrictions apply to the availability of these data, which were used under license for this study. Data are available from the authors with the permission of Australian Commonwealth and State/Territory data custodians.
