# Supplementary material for: Weight Change and Accumulation of Chronic Conditions in Women During Reproductive Years
Source: Obesity (Silver Spring). 2025 Sep 2;33(11):2195–204. doi: 10.1002/oby.70023 (PMC12559782; doi:10.1002/oby.70023)
Supplement: Supplementary file 1 — Data S1: Supporting Information. [file OBY-33-2195-s001.pdf]

Supplementary Materials

Sources of data and coverage periods

Hospital and emergency department data were obtained from each State and Territory, with some variations in coverage dates between jurisdictions. Data on investigations and procedures (e.g., glycosylated haemoglobin tests or angioplasty) were obtained from the universal health insurance scheme, Medicare (for items listed on the Medical Benefits Schedule, MBS). Similarly, data on government-subsidised medications were obtained from the Pharmaceutical Benefits Scheme (PBS) which is available to all residents. Additional data on diagnoses were available from various assessments for government supported aged care. The causes of death, including underlying and contributing causes were obtained from multiple causes coded data.

The mental health included affective disorders depression or anxiety. The cancer data only included malignant cancers, so no skin cancer is included except for melanoma. Heart disease included heart surgery and interventions (heart bypass, angioplasty, and angiography) and acute coronary syndrome, but not heart failure. Diabetes included type 1 and type 2 diabetes mellitus and excluded gestational diabetes. The asthma dataset was selected especially for asthma. The COPD data included chronic bronchitis. The fibroids only included uterine fibroids. Stroke included ischemic and haemorrhagic stroke. Dementia data set included Alzheimer’s Dementia, Vascular Dementia, and Unspecified Dementia. The eating disorders data was selected using criteria from on the multi-morbidity report for eating disorders in mental health conditions, extended with survey indications for eating disorders. Musculoskeletal disorders included back pain, rheumatoid arthritis, osteoarthritis, ankylosing spondylitis, cervical disc displacement, sciatica, spinal deformities, and scoliosis.

Table S1: List of conditions and data sources used (with coverage period) to identify patients

	Cause of Death 1996 to 2019	MBS[1] 1984 to 2021	PBS[2] 2002 to 2021	Hospital admissions/ emergency[3], varied dates	Aged care[4], varied dates	ALSWH[5] 1996 to 2019	Cancer registry
Mental health	✓	✓		✓	✓	From survey 2 onwards	
Cancer	✓	✓ From 2018		✓ From 2018			✓
Ischemic heart disease	✓	✓	✓	✓	✓	✓	
Diabetes	✓	✓	✓	✓	✓	✓	
Asthma	✓	✓	✓	✓		✓	
COPD	✓		✓	✓	✓	From survey 2 onwards	
Fibroid	✓	✓	✓	✓		✓	
Stroke	✓			✓	✓	✓	

Dementia	✓		✓	✓	✓	From survey 7 onwards	
Eating disorders	✓	✓		✓		✓	
Musculoskeletal disorders	✓		✓	✓	✓	✓	
Endometriosis		✓	✓	✓		✓	

[1] MBS: Medicare Benefits Schedule; [2] PBS: Pharmaceutical Benefits Scheme; [3] coverage of hospital data varied between States and Territories, starting between 1970 and 2007, and ending between 2017 and 2021; [4] Coverage of aged care data varied between schemes, starting between 1997 and 2008 and ending between 2015 and 2020.

[5]The first and last ALSWH surveys used in this study were conducted in 1996 and 2019.

Table S2: Distribution of BMI category in Surveys 1 and 3 for women with no chronic conditions at baseline survey (N=4 763)

		BMI category in Survey 3			
		Underweight	Normal	Overweight	Obesity
BMI category in Survey 1	Underweight	111 (22.9%)	288 (59.6%)	10 (2.1%)	74 (15.3%)
	Normal	0 (0.0%)	2517 (76.7%)	644 (19.6%)	121 (3.7%)
	Overweight	0 (0.0%)	120 (16.7%)	326 (45.3%)	274 (38.0%)
	Obesity	0 (0.0%)	6 (2.1%)	40 (14.4%)	232 (83.5%)
